# Animal welfare and society—Part 3, Response to Jocelyne Porcher

**DOI:** 10.1093/af/vfac007

**Published:** 2022-03-17

**Authors:** Gary L Francione

**Affiliations:** 1 Department of Law, Rutgers University Law School, Camden, NJ 08102, USA; 2 Department of Philosophy, University of Lincoln, Brayford Pool, Lincoln LN6 7TS, UK; 3 Department of Philosophy, School of Politics, Philosophy, Language and Communication Studies, University of East Anglia, Norwich, NR4 7TJ, UK

Response

Jocelyne Porcher does not disagree that our present approach to “animal welfare” treats animals as commodities and fails to provide protection for their interests. But she disagrees that the solution is to stop using animals as resources for humans. She argues instead that the solution is to substitute “respect” for “welfare” and to focus not on the conditions of using animals as commodities but on providing “good working and living conditions for working animals.” She rejects industrial farming and industrial slaughter but maintains that there is nothing morally wrong with raising and killing animals for food and other purposes as long as the animals have been treated with “respect.” I have five responses:

First, under Porcher’s approach, animals still remain as *our* property. Describing “working animals” presumes and incorporates their subjugation from the outset and begs every related question. *We* can still use and kill *them* for our purposes. *We* still get to decide how to value *them*. The difference is that she maintains that we should accord animal interests a higher value that represents our “respect” for animals. She proposes, in effect, what I call “happy exploitation.” We presently have “higher welfare” products available that are not much better than conventional products in terms of “humane” treatment but are *much* more expensive. What Porcher is proposing would increase the cost many times and would, in effect, mean that only the wealthy could afford these products. In a world in which animal use was still widely accepted morally, it is difficult to see how this could be possible legally, economically, or politically. Indeed, it would be rejected as elitist.

Second, analogizing animals that we use and kill for food and other purposes to coworkers in a production process ignores that a labor relationship involving humans involves *persons*—beings who have a morally and legally significant interest in continuing to live and having other fundamental interests respected. Any “labor relationship” involving nonhuman animals *necessarily* involves human persons who, as property owners, have rights in nonhuman *things* that are used and killed for humans.

In her discussion of milk and milking, she ignores that *all* systems of dairy production involve continually impregnating a cow and taking her calf away from her more or less shortly after birth. I have witnessed the separation of cow from calf on numerous occasions, including on supposedly “higher welfare” farms, and the separation process is profoundly traumatic for both. The cow will eventually be slaughtered, usually after about 6 yr. The male calves will be killed immediately or sold for veal. The idea that *however* this done is consistent with “respect” for the animals involved, or that it can be described plausibly as involving a labor relationship rather than exploitation of vulnerable beings who have no say in the situation, is something with which I disagree profoundly.

Third, Porcher argues that “respect” for animals is consistent with eating them and products made from them. She notes that we have been consuming animals for many thousands of years, but that carries no moral weight whatsoever. We have had rampant misogyny for at least that long. Doing something for a long time does not make it right.

Porcher’s primary argument about killing and eating animals is that we do nothing wrong as long as we have raised them with as much respect as possible. She talks about the care that she provides for her animals and that farmers, like her, who “respect” their animals are “at the service of their animals” all day and night and provide the “best possible life.” That, of course, does not address the moral issue here. Some slave owners said the same things about their slaves: they decried the harsh treatment of slaves by other slave owners and maintained that the relationship of a master to slave required “respect” and recognition of the productive relationship in which both shared. No doubt Porcher would reject this analogy and claim that human and nonhuman animals are different. That is, of course, true, but the only similarity necessary to rule out both animal agriculture and human slavery is sentience. As I discussed, if a being is sentient, that being *necessarily* has an interest in continuing to live. Porcher’s claim that the nonhuman situation is different from slavery on that basic level is an expression of anthropocentrism—nothing more. It is only because most of us have a vested interest in exploiting animals that we don’t see this.

Porcher argues that if we “separate” the connections between hens and eggs, chickens and poultry, etc., “the meaning disappears and eating animals seems an unnecessary cruelty.” I confess that I am bewildered by this argument. In any event, it is clearly not necessary in any sense to eat animal foods so that the production of animal foods involves unnecessary harm with or without “connections.” And any use that does not involve a conflict or compulsion *cannot* be “respectful.”

Fourth, Porcher suggests that we may come to see plants and sentient and then we will be morally obligated to starve. There is *no* scientific evidence that plants have *any* sort of mind that prefers, desires, or wants to continue to live. And, if plants were sentient, given that it takes many pounds of plants to produce one pound of meat, we would be still morally obligated to eat the plants directly if we resolved not to starve. If we all were vegan, we would reduce the amount of land necessary to grow crops to feed animals by the size of the continent of Africa. So I don’t think her deflection to the plant argument need worry us. 

Fifth, Porcher argues how different species have different interests and that according to equal consideration to them in terms of “respect” will require differential treatment. I do not dispute that different species of animal have different interests. But I do not think that, as a moral, matter, we can accord animals equal consideration because animals are property and their interests will *always* count for less.

## About the Author

Gary L. Francione is Board of Governors Distinguished Professor of Law and Nicholas deB. Katzenbach Scholar of Law and Philosophy at Rutgers University Law School, Visiting Professor of Philosophy at the University of Lincoln (UK), and Honorary Professor of Philosophy at the University of East Anglia (UK). His most recent book is Why Veganism Matters: The Moral Value of Animals.

